# Associations of adolescent mental health and parental education with healthcare use: a cohort study based on data from the Young-HUNT study, Norway

**DOI:** 10.1136/bmjment-2024-301508

**Published:** 2025-06-26

**Authors:** Kirsti Wahlberg, Karoline Louise Imingen Selvik, Tonje Braaten, Kirsti Kvaløy, Gunnhild Åberge Vie, Ottar Bjerkeset, Kristine Pape

**Affiliations:** 1 Department of Public Health and Nursing, Norwegian University of Science and Technology, Trondheim, Norway; 2 Faculty of Nursing and Health Sciences, Nord University, Bodø, Norway; 3 Department of Mental Health and Addiction Medicine, Nordland Hospital Trust, Bodø, Norway; 4 Department of Community Medicine, UiT The Arctic University of Norway, Tromsø, Norway; 5 HUNT Research Center, Department of Public Health and Nursing, NTNU Norwegian University of Science and Technology, Trondheim, Norway; 6 Levanger Hospital, Nord-Trøndelag Hospital Trust, Levanger, Norway; 7 Centre for Sami Health Research, Department of Community Medicine, Faculty of Health Sciences, UiT The Arctic University of Norway, Tromsø, Norway; 8 General Practice Research Unit, Department of Public Health and Nursing, Norwegian University of Science and Technology, Trondheim, Norway

**Keywords:** Data Interpretation, Statistical, Depression, Anxiety disorders, Child & adolescent psychiatry

## Abstract

**Background:**

Adolescent mental health problems and mental health help-seeking have increased in the later decades. We aimed to assess to which extent adolescents with high mental health symptom loads received help in general practice or specialist mental health services, and whether parental education influenced the association.

**Methods:**

This cohort study included 7554 Norwegian adolescents who participated in the population-based Young-HUNT4 Survey (2017–2019). They reported their mental health by the Strengths and Difficulties Questionnaire (SDQ), measuring both internalising and externalising symptoms, summed up to a Total Difficulties score. During 1 year after participation, data on contacts with and diagnoses from general practice and specialist mental health services were collected from national registries. We used generalised linear models to analyse the probability of contact with general practice and specialist mental health services by low, moderate and high SDQ scores. We evaluated effect measure modification using interaction terms.

**Results:**

Among adolescents with high total, internalising or externalising SDQ scores, 29–31% of females and 19–21% of males consulted a general practitioner for mental health problems, compared with 9–10% and 6–7% among those with low scores. Males and females with high internalising scores had a nine times increased risk of specialist mental health service contacts with internalising diagnoses, and similar associations were found for externalising scores and contacts/diagnoses. The associations were largely independent of parental education level.

**Conclusion:**

Adolescents’ mental health symptom loads were positively associated with health service use in general practice and specialist mental health services and largely independent of parental education.

**WHAT IS ALREADY KNOWN ON THIS TOPIC**

- Nordic studies have shown varying equitability in the utilisation of child and adolescent mental health services for adolescents according to symptom load.
- To our knowledge, a study of these interactions in both primary and secondary services in a wider age range was lacking.

**WHAT THIS STUDY ADDS**

- Norwegian adolescents with high symptom load receive help and treatment from both general practice and child and adolescent mental health services, regardless of parental education level.

**HOW THIS STUDY MIGHT AFFECT RESEARCH, PRACTICE OR POLICY**

- Though we point to the fact that adolescents seek help in general practice and specialist mental healthcare, additional information on other low-threshold services would widen our knowledge on the help-seeking patterns of adolescents with mental health symptoms.

## Introduction

 Adolescents report increasing mental health problems, particularly in the last two decades, both in Norway and other western countries.^1 2^ The most common disorders in this age group are internalising disorders such as anxiety and depressive disorders, and externalising disorders such as attention deficit hyperactivity disorder (ADHD) and oppositional defiant disorder.^3^ Whereas externalising disorders are more frequently diagnosed among male children and younger adolescents, internalising disorders are typically seen among adolescent females.^3 4^ A high externalising or internalising symptom load increases help seeking and healthcare use, and externalising symptoms might be more easily recognised by schools or parents and therefore also have more impact on healthcare contacts.^4^

Healthcare use for mental health problems has increased among adolescents in Norway, including general practitioner (GP) consultations over the last two decades^5^ and outpatient contacts in child and adolescent mental health services (CAMHS) between 2013 and 2017.^6^ US time trend studies in this period show stable or increasing adolescent mental healthcare use.^7 8^ Increased use could arise from poorer mental health,^1 2^ changes in perceived barriers to mental healthcare seeking^9^ and political focus on mental healthcare awareness and availability.^10^ In Norway, GP consultations are free of charge until age 16,^11^ while CAMHS are free until age 18.^12^ In Norway, there are 5.2 practicing physicians per 1000 population, which is higher than the OECD (The Organisation for Economic Co-operation and Development) average.^13^ All registered citizens have the right to have a regular GP, while contacts with specialist mental healthcare services (MHS) require referrals.^11^

Equal access to healthcare is an important value in the Norwegian healthcare system.^11^ There is a well-known social gradient in adolescent mental health, particularly for externalising disorders.^14 15^ A systematic review found that parental education or household income did not predict offspring mental health help-seeking.^16^ Further, a Norwegian registry linkage study of adolescents aged 16–19 found largely equitable (ie, fair) utilisation of CAMHS.^17^ However, findings from a Swedish study on adolescents aged 13–14 suggest poorer equitability for internalising than externalising symptoms.^18^ Few Nordic studies have examined the association between self-reported mental health and socioeconomic status (SES) on mental health contacts in primary and secondary healthcare among both younger and older adolescents.

We aim to assess the use of general practice and specialist MHS, including diagnostic groups, among adolescent males and females according to their levels of self-reported mental health, and whether this relationship differed with SES measured by level of parental education.

## Methods

### Setting

The Young-HUNT4 Survey was primarily school-based and performed between August 2017 and January 2019 in the northern part of Trøndelag county, Norway.^19^

### Study design

Participant responses were linked to national registers. The health service use observation period was from the registered Young-HUNT4 Survey participation date until 365 days after participation.

### Participants

Adolescents attending lower or upper secondary schools completed the survey online during school hours. Apprentices and adolescents who did not attend school on the day of the examination received the survey by post. Age was primarily 13–19; however, due to the school setting, some participants were aged 12.^19^ Five participants were not observed throughout the whole year and were excluded. See online supplemental figure 1 for exclusions.

### Data sources/measurement

Self-reported mental health problems were collected from the Young-HUNT4 Survey, while background data on sex, parental education and immigration status, as well as residency and deaths, were obtained from Statistics Norway. Information on healthcare consultations with respective diagnoses from primary care was available from the Control and Payment of Health Reimbursement Register (KUHR), and secondary care contacts with diagnoses from the Norwegian Patient Registry.

### Variables

#### Exposure variables

Mental health symptoms were measured with the Strengths and Difficulties Questionnaire (SDQ), a validated and widely used screening instrument with good psychometric properties, used both as a clinical and a research tool.^20^ Participants answered the 25-item self-report version for adolescents, asking them to consider how things have been over the last 6 months (alternatives not true, somewhat true or certainly true, scoring 0–2).^21^ SDQ measures psychological adjustment and psychopathology in adolescents, by five subgroups (emotional, conduct, hyperactivity-inattention, peer problems and prosocial).^20^ If three or more questions per subgroup of five were answered, we calculated the average score for that subgroup and multiplied by five.^21^ We summarised these into an internalising (SDQ-int, emotional and peer problems) and an externalising (SDQ-ext, conduct and hyperactivity-inattention) subscore^22^ in addition to the Total Difficulties score (SDQ-total, all except prosocial). We set cut-offs for SDQ scores based on the 80th and 90th percentiles^23^ of our own study sample (<8 low, 8–9 moderate and >9 high for both SDQ-int and SDQ-ext, and <15 low, 15–17 moderate and >17 high for SDQ-total).

#### Outcome variables

The main outcome measures are (1) general practice mental health consultations and (2) specialist mental healthcare service contacts during 1 year after survey participation.

#### General practice

We identified any consultation (yes/no), including consultations (also electronic) at GP or out-of-hours offices, and home visits. Any consultation with mental health diagnoses (yes/no) comprised consultations with any symptom or diagnosis code from the P chapter (except P27) in ICPC-2 (International Classification of Primary Care, 2nd edition).

#### Mental health services

We defined any contact (yes/no) as any outpatient contact or inpatient admissions to specialised adult or child and adolescent mental health services including interdisciplinary specialised addiction treatment. In addition, we identified any contact (yes/no) for two diagnostic subgroups based on ICD-10 (the International Statistical Classification of Diseases and Related Health Problems 10th Revision) codes: internalising and externalising mental disorders (see online supplemental table 1). It is possible to register several diagnoses per contact, thus one consultation/contact could contribute to more than one diagnostic subgroup.

#### Covariates

SES was measured by level of parental education the year the participant turned 16. It was dichotomised to higher (one or both parents with more than upper secondary schooling) and lower (both parents maximally completed upper secondary schooling).

When adjusting for age, we used participation age as registered at baseline in the Young-HUNT4 Survey. In an additional analysis, we stratified participants into over or under 16 years. Immigration status was dichotomised to ‘Norwegian-born to Norwegian parents’ or ‘other’.

### Statistical methods

We performed generalised linear model analyses with a binomial distribution and log link using complete cases. We used Stata V.17 and 18 for the analyses, and we used R for figure design.

In the main analyses, we examined the association between symptom level (SDQ score category) and healthcare use. We adjusted for level of parental education, age and immigration status, and stratified by sex. Analyses were repeated for each healthcare outcome. We estimated relative risks and postestimation probabilities, and results are shown with 95% CIs.

Similar analyses were performed to assess socioeconomic differences, but we now introduced an interaction term between symptom level (SDQ score) and level of parental education, adjusted for age, sex and immigration status. We tested the hypothesis of no interaction by parental education level on the relationship between SDQ symptom load and health service use by using a joint Wald test. Joint Wald tests were also used for the evaluation of statistical interaction between SDQ symptom load and sex (adjusted for parental education, age and immigration status) and age over 16 (yes/no, adjusted for parental education and immigration status and stratified by sex).

As sensitivity analyses, we assessed associations between missing SDQ scores (all missing scores) and healthcare use. As an alternative measure to estimate the approximate length of treatment, we tabulated the number of months of contact over the observed year.

## Results

### Descriptive results

The Young-HUNT4 Survey sample consisted of 7554 youth born 1997–2006, with 51.4% females. Participation age ranged from 12.7 to 19.9 years, and 87.9% were born in Norway to Norwegian parents. Further, 40.3% had low SES (0.6% missing), see table 1. More females than males had high symptom scores on SDQ-total (14.6% vs 8.0%) and SDQ-int (16.9% vs 5.9%), but not on SDQ-ext (10.8% vs 13.2%). Invalid scores for SDQ-total, SDQ-int and SDQ-ext were found in 16, 6 and 10 people, respectively, in addition to the 494 excluded for all SDQ scores being invalid. Of those with a high SDQ-ext, 31% also had a high SDQ-int, and 32% of those with a high SDQ-int also had a high score on SDQ-ext. Overall, 71.5% (any consultation) and 10.9% (mental health consultation) consulted a GP, while 7.5% had been in contact with MHS.

**Table 1:** Demographics of the final sample of participants

	Females	Males	Total
N	3886	3668	7554
Females			51.4
Age mean (SD)	16.1 (1.8)	16.1 (1.8)	16.1 (1.8)
High SES	58.8	59.4	59.1
SES missing	0.4	0.7	0.6
Norwegian-born to Norwegian parents	87.9	87.9	87.9
Immigration status missing	0.0	0.0	0.0
SDQ-total			
Low	71.7	83.5	77.4
Moderate	13.5	8.3	11.0
High	14.6	8.0	11.4
Missing	0.3	0.2	0.2
SDQ-int			
Low	69.5	86.7	77.8
Moderate	13.6	7.3	10.5
High	16.9	5.9	11.6
Missing	0.1	0.1	0.1
SDQ-ext			
Low	74.4	72.3	73.4
Moderate	14.6	14.4	14.5
High	10.8	13.2	11.9
Missing	0.2	0.1	0.1
Healthcare use n(%)			
Any GP consultation	2999 (77.2%)	2402 (65.5%)	5,401 (71.5%)
GP mental health	515 (13.3%)	305 (8.3%)	820 (10.9%)
Any MHS	403 (10.4%)	162 (4.4%)	565 (7.5%)
No observed contact	843 (21.7%)	1223 (33.3%)	2,066 (27.4%)

Number in percentage (%) where not otherwise specified.

GP, general practitioner; MHS, mental healthcare services; SDQ, Strengths and Difficulties Questionnaire; SDQ-ext, SDQ externalising; SDQ-int, SDQ internalizing; SES, socioeconomic status.

### Main results

In general, there was a clear positive dose-response relationship between elevated SDQ symptom scores and healthcare contacts during the year after survey participation for both males and females. This applied to the total, internalising and externalising SDQ symptom scores and was evident across all types of healthcare contacts. Of adolescents with high SDQ-total scores, the majority contacted either GP or MHS. Further, 28% of males and 13% of females with high SDQ-total did not have any contact with GP or MHS (online supplemental table 2).

### General practice

**Figure 1:**
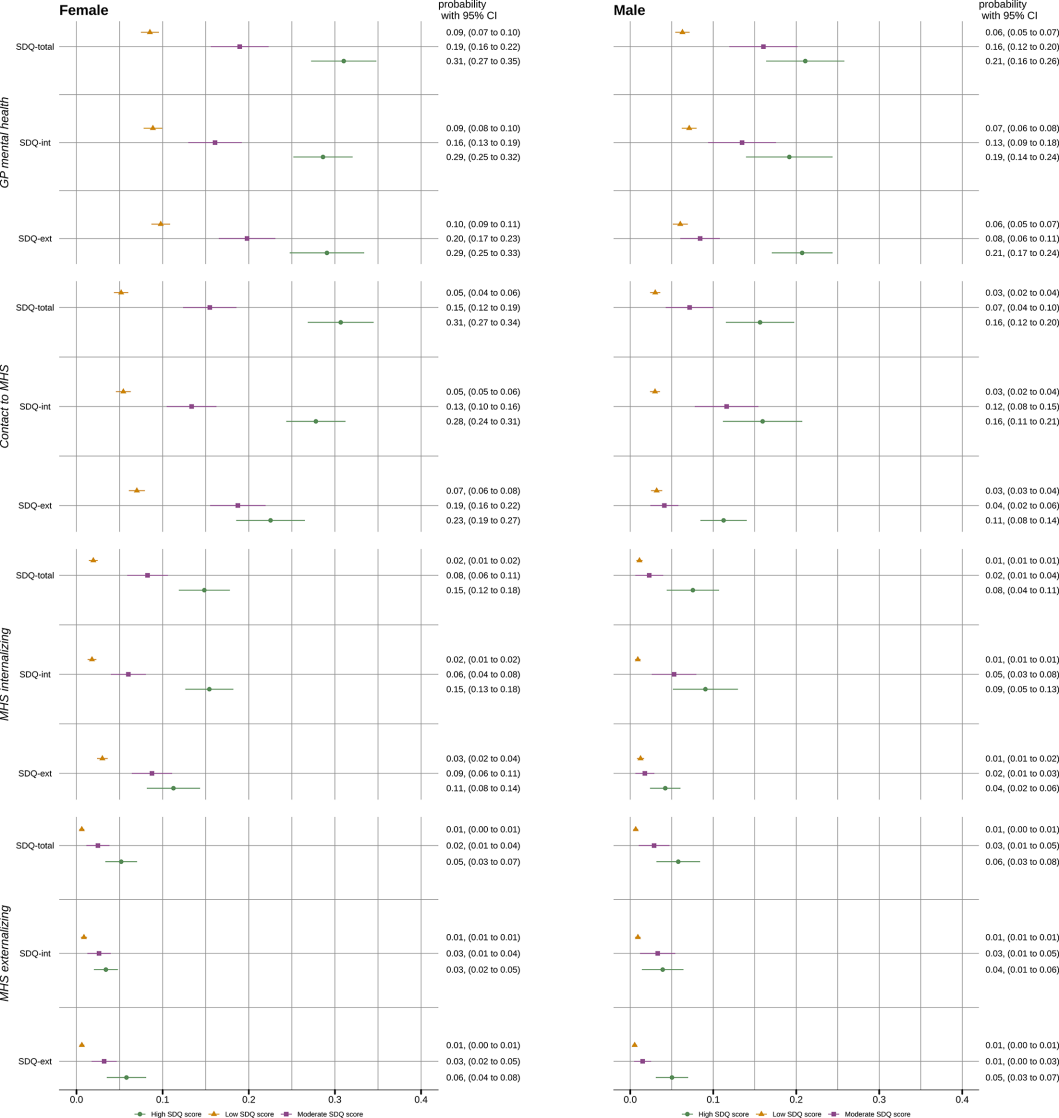
Estimated probability of mental health consultations in general practice (GP) or mental health services (MHS) contacts, including contacts with selected diagnosis groups. By level of total (SDQ-total), internalising (SDQ-int) and externalising (SDQ-ext) score on the Strengths and Difficulties Questionnaire (SDQ). Presented separately by sex. Based on a generalised linear model adjusted for age, parental education level and immigration status.

For females with a high SDQ-total score, estimated probability of mental health consultations in general practice was 0.31 (95% CI 0.27 to 0.35), compared with 0.19 (95% CI 0.16 to 0.22) for those with a moderate and 0.09 (CI 0.07 to 0.10) for those with a low score (figure 1). Corresponding numbers for males were 0.21 (95% CI 0.16 to 0.26), 0.16 (95% CI 0.12 to 0.20) and 0.06 (95% CI 0.05 to 0.07), respectively. The patterns were similar for internalising and externalising subscores.

**Figure 2:**
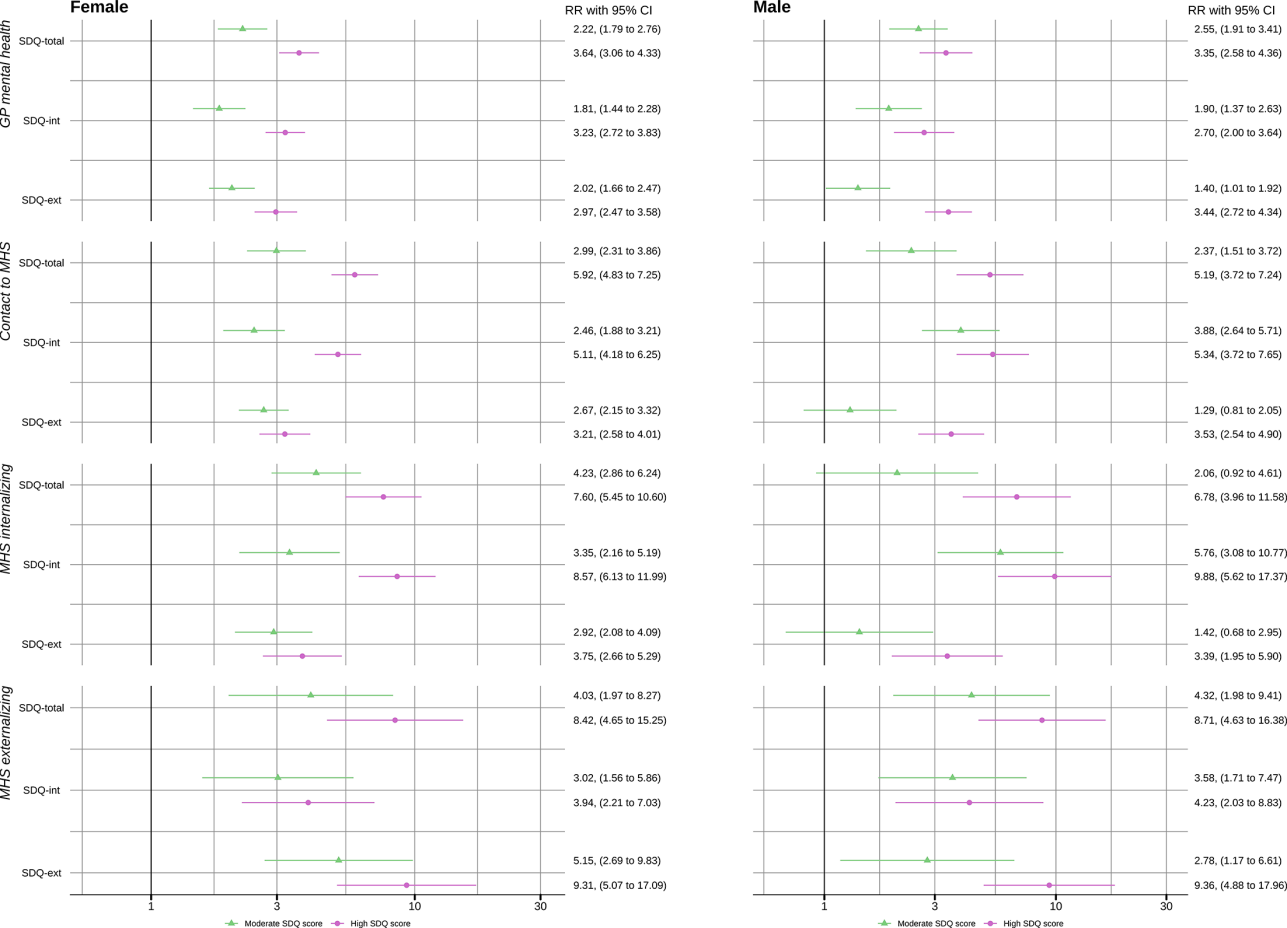
Relative risks (RRs) of mental health contacts to general practice (GP) or contacts to mental health services (MHS), including contacts with selected diagnosis groups. By level of total (SDQ-total), internalising (SDQ-int) and externalising (SDQ-ext) score on the Strengths and Difficulties Questionnaire (SDQ), compared with participants with low scores. Presented separately by sex. Based on a generalised linear model adjusted for age, parental education level and immigration status.

Figure 2 presents the corresponding relative risks. Compared with low SDQ scores, males and females with high scores (SDQ-total, SDQ-int and SDQ-ext) had a 2.7–3.6 times higher risk of GP mental health consultations. Differences in associations between males and females were small (joint Wald tests for SDQ-int and any MHS contact p=0.02, joint Wald test for other associations p=0.04–0.6).

### Mental health services

Overall, females had higher estimated probabilities of contacts to MHS than males, except for contacts with externalising diagnoses. Females with high SDQ-int scores had an estimated probability of 0.15 (95% CI 0.13 to 0.18) to have a contact with an internalising diagnosis, compared with 0.02 (95% CI 0.01 to 0.02) for females with normal SDQ-int scores (figure 1). This corresponds to a ninefold increased risk of contact (relative risk (RR) 8.6, 95% CI 6.1 to 12.0), see figure 2. Corresponding probabilities for males were 0.09 (95% CI 0.05 to 0.13) and 0.01 (95% CI 0.01 to 0.01), with RR 9.9 (95% CI 5.6 to 17.4). Similarly, females with a high SDQ-ext score had a probability of 0.06 (95% CI 0.04 to 0.08) and males 0.05 (95%CI 0.03 to 0.07) for an MHS contact with an externalising diagnosis, in contrast to 0.01 (95% CI 0.00 to 0.01) for both females and males with normal SDQ-ext. Relative risks for high SDQ-ext for contacts with externalising disorders were 9.3 (95% CI 5.1 to 17.1) for females and 9.4 (95% CI 4.9 to 18.0) for males. The association between moderate SDQ-ext and any MHS contact was weaker for males compared with females (joint Wald test p=0.01). Other associations between SDQ levels and MHS contacts were largely similar among males and females (joint Wald tests p=0.15–0.96).

### Parental education level

**Figure 3:**
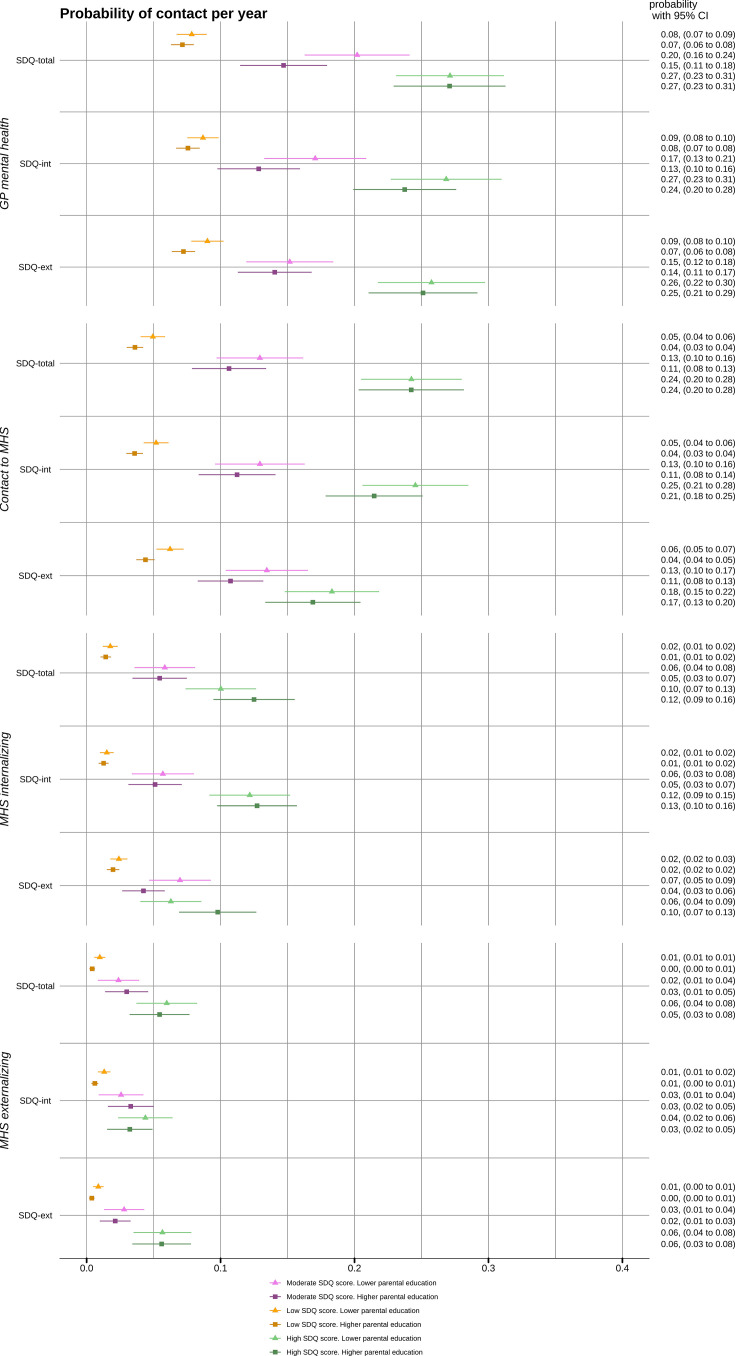
Estimated probability of mental health consultations in general practice (GP) or mental health services (MHS) contacts, including contacts with selected diagnosis groups. By level of total (SDQ-total), internalising (SDQ-int) and externalising (SDQ-ext) score on the Strengths and Difficulties Questionnaire (SDQ) and parental education (dichotomised as one or both parents having more than upper secondary schooling vs both parents having upper secondary schooling or less) as a measure of socioeconomic status. Based on a generalised linear model adjusted for age, sex and immigration status.

Generally, patterns of increased healthcare use for adolescents with high symptom load were consistent regardless of parental education level, as confirmed by joint Wald tests for most associations ranging from 0.09 to 0.72. The probabilities of healthcare contacts by contact type, SDQ score and parental education are shown in figure 3.

For SDQ-ext, the probability of contact with internalising diagnoses was not higher for those with high SDQ-ext than those with moderate if they had lower SES (estimated probabilities 0.06, (95% CI 0.04 to 0.09) and 0.07, (95% CI 0.05 to 0.09), respectively). The corresponding probabilities among those with high SES were 0.07 (95% CI 0.05 to 0.09) and 0.10 (0.07–0.13), joint Wald test p=0.02. However, the proportion with no observed contact also tended to be slightly lower among those with lower SES and moderate or high SDQ-total (online supplemental table 2).

### Additional analyses

Overall, effect sizes were similar before and after age 16 (online supplemental figures 2 and 3). However, in contrast to females younger than 16, RRs for any contact to MHS were not higher with high SDQ-ext than with moderate SDQ-ext for females age 16 and above (joint Wald tests p=0.03).

Male survey participants lacking SDQ scores had more GP consultations with mental health diagnoses and MHS contacts with external diagnoses compared with males with complete information (online supplemental figure 4). Number of months with GP mental health contacts and MHS contacts was also higher with higher SDQ-total score (online supplemental table 3).

## Discussion

### Key results

In this 12-month follow-up of 7554 Norwegian adolescents, we found higher mental health service use among males and females with elevated total, internalising and externalising SDQ symptom scores. This was found both in GP mental health consultations and in specialist MHS, and for different mental health diagnosis groups. We did not find considerable social inequity by level of parental education in the association between SDQ score and health service use.

### Strengths and limitations

The Young-HUNT4 Survey has a high participation rate,^19^ and linkage with a national registry gives complete and valid information on health service use and diagnosis in both primary and secondary healthcare,^24^ avoiding recall and social desirability bias. Further, the SDQ instrument gives insights into both internalising and externalising symptoms. A limitation of using cut-offs based on our sample is that the non-participants might have higher symptom loads, leading to higher symptom load category in our sample compared with a hypothetical complete sample. However, the total score percentiles in our sample corresponded well with previous studies.^23^ In terms of diagnostic classification, we chose wider diagnosis groups to account for the variability in the diagnosing in MHS and between healthcare workers.

The survey is performed in a mostly rural area, and the cohort might thus not be representative of larger city populations,^19 25^ for example, regarding ethnical diversity.^16 19^ However, the Young-HUNT4 Study is fairly representative of Norwegian adolescents. As this survey is primarily performed at schools, absent adolescents are under-represented, which may have led to the underestimation of healthcare use.^19^ Few participants were missing data; however, our additional analyses suggested boys who did not complete the SDQ questionnaire in the Young-HUNT4 Study had more MHS contacts with externalising diagnoses.

To get a better estimate of need for care, we would normally consider symptom load and function level together. However, the SDQ impact scale or other appropriate measures of function were not available. Performing many analyses increases the risk of type I error. Therefore, we have interpreted our results with caution. Another disadvantage is our lack of registry data on other types of health services, including municipal youth health centres and private medical services without public reimbursement agreement, though very few fully private mental healthcare services exist for children and adolescents in Norway. Information on these services would identify other potential sources of help and give a better estimate of the adolescents’ help-seeking patterns.

Different indicators for SES might impact health and health service use differentially. A systematic review found that household income and parental education affected children and adolescents’ mental health more strongly than parental occupational status.^14^ In the context of the Norwegian welfare scheme, we expect parental education to capture important aspects of SES for adolescents. However, different measures or different categorizations could have revealed different results.

We have chosen to dichotomise our outcomes, as the exact number of contacts to MHS cannot be distinguished in our material. We thus avoid overestimating the number of contacts; however, we lose information on contact frequency and exact length of treatment. As indicated by our additional analyses, the treatment duration is also driven by higher symptom load.

### Interpretation

Positive associations between SDQ symptom load and healthcare use correspond well to earlier findings from Norway and Germany.^26^,^28^ These papers used self-reported (or parent-reported) healthcare use, and the Norwegian studies screened for internalising symptoms only.

While a majority of adolescents consult a GP within a year, only 21% (males) and 31% (females) of those with a high SDQ total have a GP consultation with a mental health diagnosis. Thus, many adolescents with considerable mental health symptoms do not seek their GP for this problem, although some of them might have presented with psychosomatic symptoms. Sickness certification requirements for upper secondary school students increased the frequency of GP consultations in the study period, though less so for mental health diagnoses.^29^ For participants with low SDQ-total, around a tenth had a mental health consultation with GP or in MHS.

Our finding that higher contact frequency for females than males in both GP and MHS,^5 17^ except for externalising diagnoses, is in keeping with previous research.^3^

Earlier literature found no clear association between parental education and health services use for mental health problems,^16^ though living in an area of higher SES is one of the determinants of MHS use.^25^ Similar to the findings from Western Norway, we found no clear socioeconomic differences, measured by parental education, in how symptom load affects healthcare use in MHS.^17^ A Swedish study, however, found lower specialist mental healthcare use for females with lower parental education and high symptom load, except for ADHD/autism.^18^ Our study widens the knowledge on this topic by including general practice and analysing a sample with a wider age group.

We found no major differences between levels of parental education, except for the probability of contact with internalising diagnosis for participants increasing SDQ-ext scores. A German study found SES to be a predictor of any service use but not for mental health services use, though mental health services were somewhat differently defined.^27^ A Norwegian survey among physicians showed that doctors over the period 2008–2016 became more likely to adapt their treatment according to patients’ socioeconomic factors, for example, by using more time.^30^ Thus, doctors might contribute to social equity in healthcare. In addition, adolescents with higher SES might have more knowledge of or economic ability to seek help, also elsewhere, as in private psychologists or similar. Data on private providers or from youth health centres would broaden our knowledge of socioeconomic differences in the use of these primary providers. In total, there are few signs of a considerable effect of SES on the relationship between symptom load and health services use in Norwegian adolescents.

## Conclusion

We found an increasing healthcare use with increasing SDQ symptom load, both in GP office mental health consultations and in MHS contacts including mental health diagnosis groups. SES, measured by parental education, had little effect on the association between SDQ symptom load and healthcare use. We argue that Norwegian adolescents with high symptom load receive help and treatment from both GP and MHS, regardless of parental education level.

## Supplementary material

10.1136/bmjment-2024-301508 online supplemental file 1

## Data Availability

Data may be obtained from a third party and are not publicly available.
