# Supplementary material for: Associations of adolescent mental health and parental education with healthcare use: a cohort study based on data from the Young-HUNT study, Norway
Source: BMJ Ment Health. 2025 Jun 26;28(1):e301508. doi: 10.1136/bmjment-2024-301508 (PMC12207154; doi:10.1136/bmjment-2024-301508)

# Supplementary materials

## Supplementary table 1

Table of classification of contacts and diagnosis groups in general practice and mental health services.

| **Health service:** | **Group:** | **Coding:** |
| --- | --- | --- |
| **General practice:** |  | **Tariff for GPs and OOH doctors** |
|  | **Any consultation** | 2ad, 2ae, 2ak, 2ed, 2fk, 2af, 11a |
|  |  | **ICPC-2 codes:** |
|  | **Mental health diagnosis** | P01-P26, P28, P29, P70-P99 |
| **Mental health service:** |  |  |
|  | **Any contact** | Any observation in MHS data |
|  |  | **ICD-10 codes:** |
|  | **Externalizing disorders** | F90-F91.9, F10-F19.9, F60.2, F60.3, F63 |
|  | **Internalizing disorders** | F30-F34.9, F38-F44.9, F93-F93.9. |

## Supplementary table 2

Descriptive statistics on demographics, divided by level of SDQ-total.

| Total difficulties score | Sex/ parental education | Adolescents contacting | | | |
| --- | --- | --- | --- | --- | --- |
|  |  | Total | No observed contact | Only GP, no mental health | Mental health at GP or MHS |
| Low | Female, n (%) | 2785 | 700 (25.1%) | 1796 (64.5%) | 289 (10.4%) |
|  | Male, n (%) | 3063 | 1052 (34.3%) | 1783 (58.2%) | 228 (7.4%) |
|  | Lower, n (%) | 2212 | 668 (30.2%) | 1326 (59.9%) | 218 (9.9%) |
|  | Higher, n (%) | 3606 | 1073 (29.8%) | 2235 (62.0%) | 298 (8.3%) |
|  | Missing, n (%) | 30 | 11 (36.7%) | 18 (60.0%) | 1 (3.3%) |
| Moderate | Female, n (%) | 525 | 69 (13.1%) | 322 (61.3%) | 134 (25.5%) |
|  | Male, n (%) | 305 | 86 (28.2%) | 163 (53.4%) | 56 (18.4%) |
|  | Lower, n (%) | 380 | 48 (12.6%) | 230 (60.5%) | 102 (26.8%) |
|  | Higher, n (%) | 443 | 105 (23.7%) | 251 (56.7%) | 87 (19.6%) |
|  | Missing, n (%) | 7 | 2 (28.6%) | 4 (57.1%) | 1 (14.3%) |
| High | Female, n (%) | 566 | 72 (12.7%) | 241 (42.6%) | 253 (44.7%) |
|  | Male, n (%) | 294 | 81 (27.6%) | 135 (45.9%) | 78 (26.5%) |
|  | Lower, n (%) | 443 | 70 (15.8%) | 198 (44.7%) | 175 (39.5%) |
|  | Higher, n (%) | 411 | 83 (20.2%) | 173 (42.1%) | 155 (37.7%) |
|  | Missing, n (%) | 6 | 0 (0.0%) | 5 (83.3%) | 1 (16.7%) |

## Supplementary table 3

Months of contact, by sex and level of SDQ-total.

| **Total difficulties score** | **GP consultation for mental health** | **Any MHS contact** |
| --- | --- | --- |
| **Females** | | |
| **Low** | **0.16** | **0.20** |
| **Moderate** | **0.38** | **0.63** |
| **High** | **0.76** | **1.46** |
| **Total** | **0.28** | **0.44** |
| **Males** | | |
| **Low** | **0.13** | **0.13** |
| **Moderate** | **0.34** | **0.30** |
| **High** | **0.42** | **0.64** |
| **Total** | **0.17** | **0.18** |

## Supplementary figure 1

Flow diagram of exclusions.


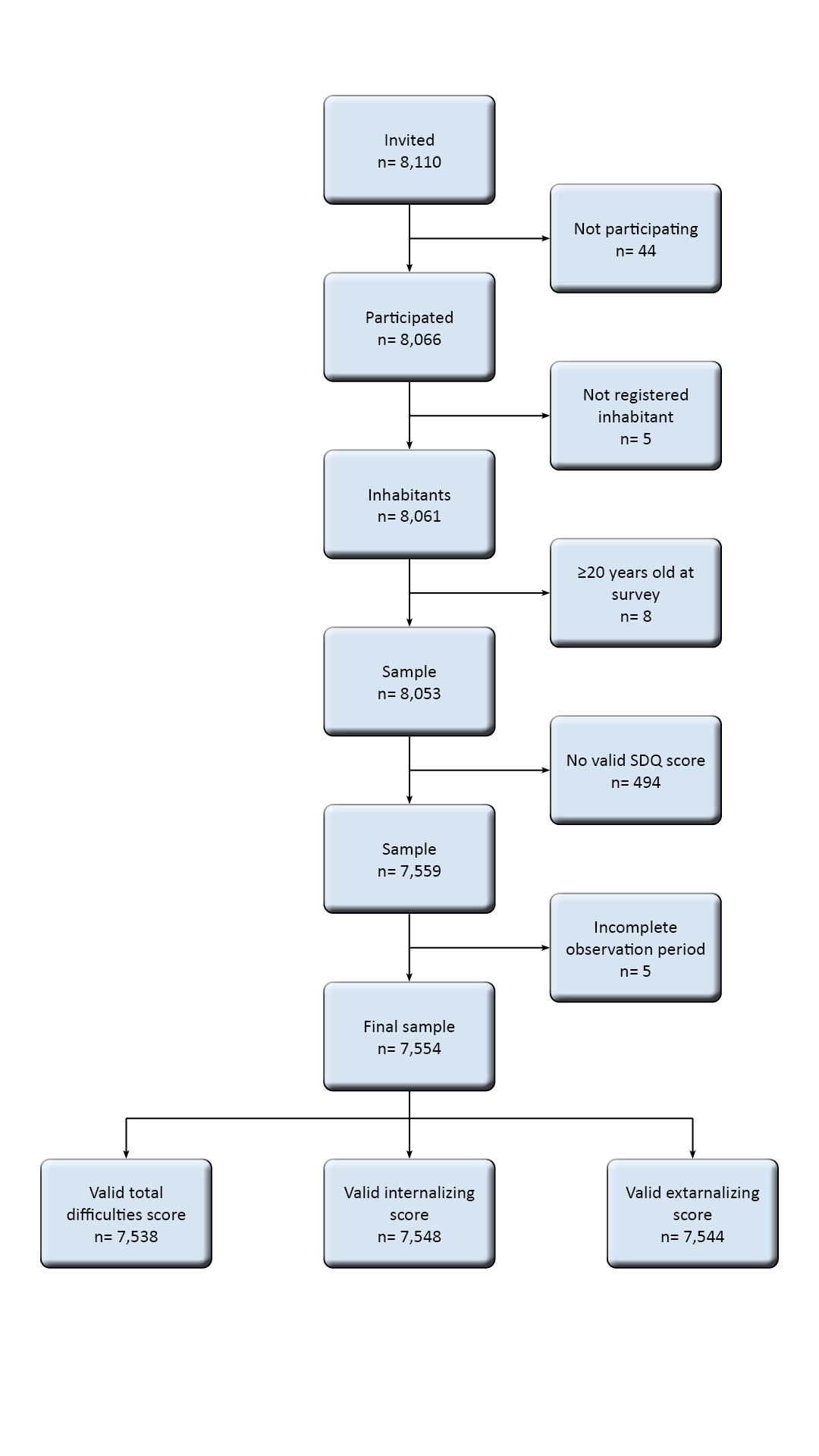


## Supplementary figure 2

Estimated probability of contacts for mental health problems to general practice (GP) or mental health services (MHS), including contacts with selected diagnosis groups. For females. By level of total (SDQ-total), internalizing (SDQ-int) and externalizing (SDQ-ext) score on the strengths and difficulties questionnaire (SDQ). Presented by age group. Based on a generalized linear model adjusted for parental education level and immigration status.


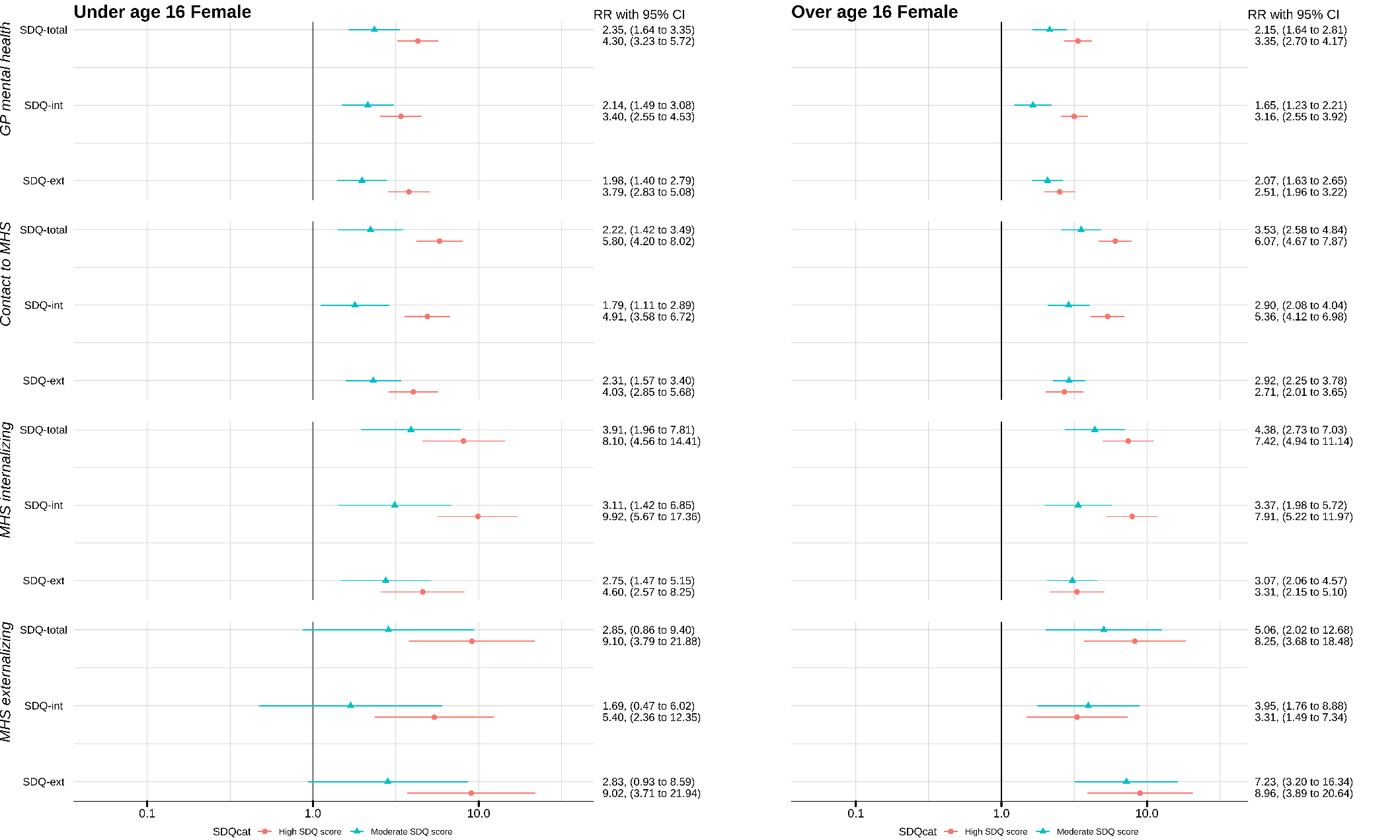


## Supplementary figure 3

Estimated probability of contacts for mental health problems to general practice (GP) or mental health services (MHS), including contacts with selected diagnosis groups. For males. By level of total (SDQ-total), internalizing (SDQ-int) and externalizing (SDQ-ext) score on the strengths and difficulties questionnaire (SDQ). Presented by age group. Based on a generalized linear model adjusted for parental education level and immigration status.


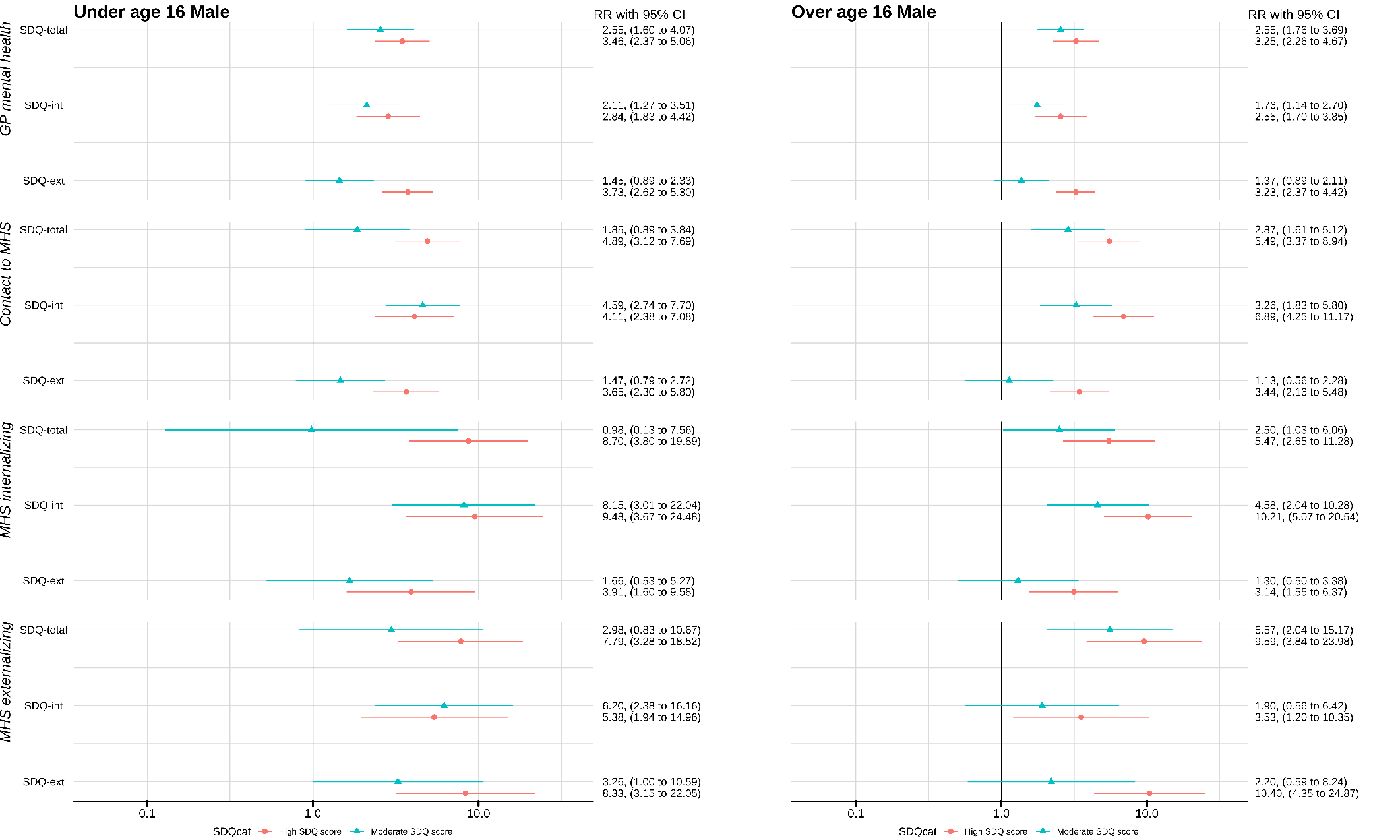


## Supplementary figure 4

Risk ratios (RRs) of mental health contacts to general practice (GP) or contacts to mental health services (MHS), including contacts with selected diagnosis groups. Ratios for missing on total (SDQ-total), internalizing (SDQ-int) and externalizing (SDQ-ext) score on the strengths and difficulties questionnaire (SDQ), compared to participants completed scores. Participants with missing on all SDQ scores included. Based on a generalized linear model, adjusted for parental education level, participation age and immigration status, stratified by sex.


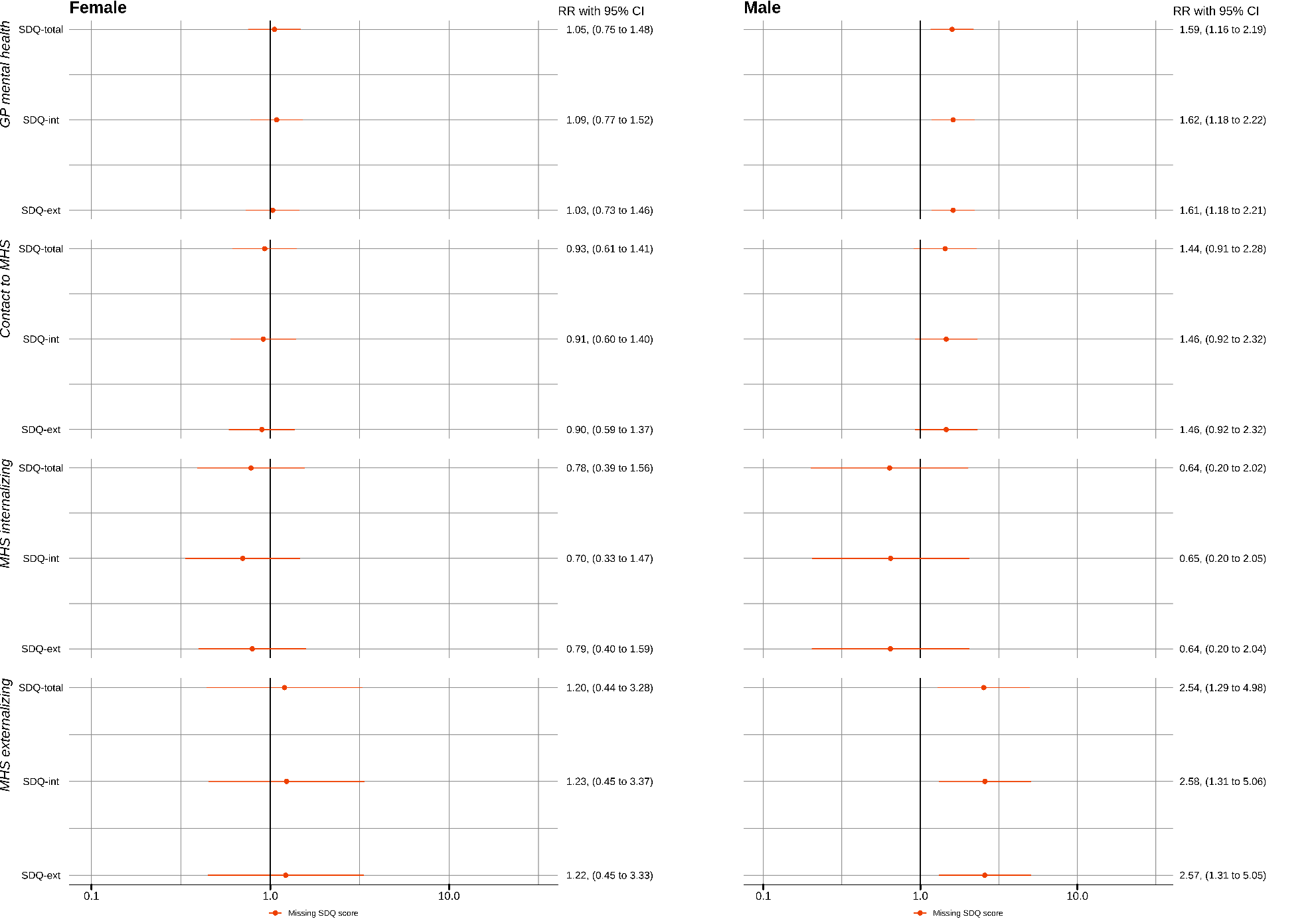

Supplement: online supplemental file 1 [file bmjment-28-1-s001.docx]
